# Ocular lamellar crystalline gels for sustained release and enhanced permeation of resveratrol against corneal neovascularization

**DOI:** 10.1080/10717544.2021.1872739

**Published:** 2021-01-21

**Authors:** Minshu Li, Xiang Yu, Lin Zhu, Yiguang Jin, Zhihong Wu

**Affiliations:** a Jinzhou Medical University, Jinzhou, China; b Department of Ophtalmology, the Third Medical Centre, Chinese PLA General Hospital, Beijing, China; c Department of Pharmaceutical Sciences, Beijing Institute of Radiation Medicine, Beijing, China; d Huzhou Central Hospital, Huzhou, China

**Keywords:** Resveratrol, lamellar liquid crystal, glyceryl monooleate, corneal neovascularization, computer simulation

## Abstract

Corneal neovascularization (CNV) is the major cause of blindness after eye injury; however, only several drugs can be applied and the invasive administration ways (i.e., intravitreal injection and subconjunctival injection) are used. Resveratrol is a highly effective anti-VEGF agent against CNV. However, its applications are limited due to its strong hydrophobicity and instability. Here, we developed a resveratrol-loaded ocular lamellar crystalline gel (ROLG) for high inhibition of CNV. ROLGs were composed of resveratrol, glyceryl monooleate (GMO), ethanol, and water, and their lamellar crystalline structures were identified by polarizing light microscopy and small-angle X-ray scattering. High drug loading (4.4 mg/g) of ROLGs was achieved due to the hydrogen bonding between GMO and resveratrol. Resveratrol showed sustained release with 67% accumulative release in 7 h, which was attributed to the slow erosion of gels. Resveratrol in ROLGs had a high corneal permeation 3 times higher than resveratrol in hyaluronic acid suspensions (RHSs). ROLGs were administered to rats only once a day because of their strong retention on the cornea surface. ROLGs were safe due to the very little contact of ethanol in ROLGs to the cornea. CNV post-rat corneal alkaline injury was highly inhibited by ROLGs, resulting from the attenuation of corneal VEGF expression and then corneal healing was improved. The ROLG was a promising ocular medicine for the prevention of CNV.

## Introduction

Corneal neovascularization (CNV), the pathological angiogenesis in corneal, is commonly caused by physical injury, infections, and chemical burns to the ocular surface (Power et al., 1998; Sharif & Sharif, 2019). CNV usually brings about corneal refractive changes that compromise visual clarity resulting in visual impairment and even blindness (Benayoun et al., 2013). Currently, intraocular injections of anti-VEGF antibodies (i.e., bevacizumab and tocilizumab) have been used as an efficient strategy to reduce CNV progression. However, direct delivery into intraocular lesions dramatically increases the risk of endophthalmitis or other injuries. Furthermore, anti-VEGF agents are too expensive to be acceptable to the general population. Therefore, there is a pressing need for an efficient and noninvasive anti-VEGF agent to treat CNV.

Resveratrol is a phytoalexin produced by vine. Its action is to stimulate the natural defense in plants. However, it also has several beneficial effects in animals and humans by its action on several organs and tissues (Burns et al., 2002; Sales & Resurreccion, 2014). It is a class II compound in the Biopharmaceutical Classification System (BCS) with high permeability (Log *P* = 3.1) and low aqueous solubility. Resveratrol has been one of the most popular small-molecule natural compounds in the pharmaceutical field due to its powerful anti-inflammatory and anti-VEGF effects. These properties have led to extensive studies for the treatment of a variety of cancers, such as breast cancer, melanomas, and visceral adipose tissue diseases (Carletto et al., 2016; Murphy et al., 2018; Poonia et al., 2019). Additionally, resveratrol has potent anti-VEGF effects and has been used successfully for the treatment of eye diseases, such as high glucose-induced retinal injury (Jiang et al., 2019; Ruginǎ et al., 2019) and corneal chemical burns (Chen et al., 2013). However, ocular resveratrol delivery is a challenge due to its high hydrophobicity, the need to permeate dense corneal epithelial layers, and its elimination through tears. Hence, it is essential to find novel ways to deliver resveratrol that is easy, effective, and safe.

Lyotropic liquid crystals have recently attracted attention for mucosal drug delivery due to their long retention time at administrated sites and high permeability across biological barriers. Lyotropic liquid crystals have been used as transdermal delivery carriers for the treatment of skin diseases. Mitoxantrone-loaded cubic lyotropic liquid crystal gels have been fabricated for the topical treatment of melanomas (Yu et al., 2016) and capsaicin transdermal cubic crystal gels have been developed to alleviate skin inflammations (Peng et al., 2010). In our preliminary studies, cubic crystals with high viscosity and low resveratrol loading were not suitable for ocular resveratrol delivery. In contrast, lamellar liquid crystals, a type of lyotropic liquid crystals, have suitable fluidity, and high drug loading (Iwata et al., 2004). In this study, we used resveratrol-loaded ocular lamellar crystalline gel (ROLG) for the treatment of CNV.

ROLG was prepared using resveratrol, glyceryl monooleate (GMO), ethanol, and water. The structures of ROLGs were determined using polarizing light microscopy (PLM) and small-angle X-ray scattering (SAXS). ROLGs with appropriate viscosity was further evaluated using a rheometer. Resveratrol crystallization was demonstrated using X-ray diffraction. The underlying molecular mechanism of the interactions between GMO and resveratrol were elucidated using molecular docking and confirmed using FTIR spectroscopy. The release mechanism was determined using *in vitro* release profiles. The effect of lamellar crystalline gels (LCGs) on resveratrol ocular permeation was investigated using *in vitro* permeation studies. Computer simulations of totally atomic molecular dynamics (MD) and dissipative particle dynamics (DPD) were used to determine the drug and ethanol distribution in the ROLGs. Furthermore, the *in vivo* properties of ROLG was investigated.

## Materials and methods

### Materials

GMO was purchased from Dansico Co., Ltd. (Boston, USA). Resveratrol (99% purity) was purchased from Sigma Aldrich Chemical Co. (Shanghai, China). Fluorescein sodium ophthalmic test paper was purchased from Jingming New Technological Development Co., Ltd. (Tianjing, China). All other materials were of analytical grade.

### Animals

Male Sprague-Dawley (SD) rats (200 ± 20 g) were purchased from the Beijing Vital River Experimental Animal Technology Co., Ltd., China. Animal handling and surgical procedures was performed according to the Guiding Principles for the Use of Laboratory Animals of the Beijing Institute of Radiation Medicine (BIRM).

### Preparation of ROLGs

The hot solution dispersing method was used for ROLG preparation (Isabelle et al., 2015). Briefly, resveratrol (42 mg) was dissolved in ethanol (0.6 g) at 55 °C. GMO (6 g) was heated to 55 °C and then mixed with the resveratrol/ethanol solution to obtain an oil phase. Hot purified water (3 g, 55 °C) was slowly introduced into the oil phase under homogeneous vortexing. The generated ROLGs were then sealed in a tube at room temperature and sent for structural identification.

### Characterization of ROLGs

The sample (0.1 g) was placed onto a glass slide and then covered with a glass coverslip. Optical images were obtained using a polarizing light microscope (PLM, DMLP, Leica, Germany).

SAXS (small-angle X-ray scattering) analysis instrument (SAXSee, Anton Paar) was used to identify the inner ROLG structure. Samples were wrapped in a thin sheet of tinfoil before measurement. Studies were performed using Ni-filtered Cu Ka radiations (1.542 Å) from a copper rotating anode operating at 45 kV and 50 mA as described in (Chen et al., 2019). Scattering intensities were plotted as a function of reciprocal spacing.

The rheological properties of ROLGs were investigated using the H-PTD200 rheometer (TA, America) equipped with a cone-plate sensor with a diameter of 20 mm, a cone angle of 1°, and a gap of 28 μm. An appropriate amount of sample was loaded onto the sample stage before the senor was slowly adjusted to attain the desired measuring gap. The measurement procedure was initiated after 2 min of equilibrium at the measured temperature. Using the oscillation-amplitude mode and a scanning frequency fixed at 1 Hz, amplitude sweep measurements were performed ranging from 0.01–100% strain to determine the linear viscoelastic region of the sample. Based on the value in the linear viscoelastic region, frequency sweep measurements were performed ranging from 0.01–100 rad/s using the oscillation-frequency mode, with the measurement temperature maintained at 32 ± 0.1 °C.

### Molecular docking

The structures of resveratrol and GMO underwent structural minimization and structural dynamic optimization using the Sybyl 6.9.1 software package (Yu et al., 2018). Complexes between resveratrol and GMO were predicted by molecular docking using the AutoDock 4.0 software. The optimized Auto Docking parameters were as follows: the maximum number of energy evaluations were 25,000,000 per run; the iterations for Solis and Wets local search were 3000; the number of generations was 100, and the number of individuals in the population was 300. Results that differed by less than 2 Å in a positional root mean square deviation were clustered together.

### Powder X-ray diffraction

Powder X-ray diffraction (PXRD) has been commonly used for the detection of crystallinity of materials and drugs (Hebert & Stöwe, 2020). Lyophilized powders of pure resveratrol, pure GMO, and GMO/resveratrol mixtures at weight ratios of 4, 8, and 16 were used to determine crystallinity. All samples were evaluated on the Powder X-ray diffractometer (Bruker, D8 Advance, Billerica, MA, USA). The voltage was set at 45 kV and the current at 40 mA. The divergence and scattering slits were set as 1°, while the receiving slit was 0.2 mm. Measurements were performed at a scan rate of 3°/min (0.4 s/0.02°) and 2*θ* scan ranging from 5° to 35°.

### Fourier transform infrared spectroscopy

Fourier transform infrared spectroscopy (FTIR) was used to validate the molecular interactions between drugs and materials (Whuisu et al., 2019). Lyophilized powders of pure resveratrol, pure GMO, and GMO/resveratrol mixtures at weight ratios of 4, 8, and 16 were measured using the FT-IR instrument equipped with an ATR tool and an MCT detector (Bruker, Ettlingrn, Germany) at wavenumbers from 4000 to 400 cm^−1^, respectively. The lyophilization process included freezing at −45 °C for 4 h, primary drying at −20 °C/0.007 mbar for 20 h, and secondary drying at 35 °C/0.0014 mbar for 40 h.

### In silico simulation of ROLGs

Simulations of totally atomic molecular dynamics (MD) and dissipative particle dynamics (DPD) were performed to determine the inner structure of the ROLGs.

**Figure 1.**
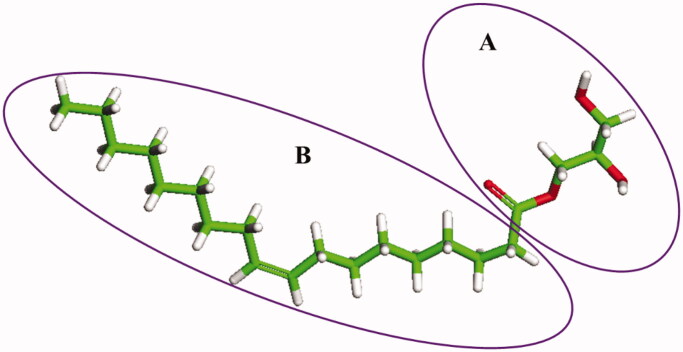
Chemical structure of GMO divided into Fragments A and B.

One GMO molecule was divided into Fragments A and B that represented the hydrophilic glycerol and carboxyl group and the hydrophobic long carbon chain, respectively (Figure 1). ROLGs were constructed using the Amorphous Cell under the COMPASS force field in Materials Studio 7.0 (Accelrys Co., US). The initial configurations were subjected to 10,000 steps of energy minimization with an energy convergence threshold of 1 × 10^−4^ ^ ^kcal/mol and force convergence of 0.005 kcal/mol/Å. Afterward, thermal annealing was performed from 1000 to 300 K. 500 ps MD equilibrium simulations of one pure component were performed under isothermal-isobaric (NPT) conditions, whose trajectory files were generated every 5 ps. The final 10 configurations were used to calculate *δ* and cohesive energy densities via the Forcite module. Similarly, binary components were subjected to the above MD equilibrium simulations under the isothermal-isochoric (NVT) condition and their trajectory files and cohesive energy densities were also generated as above. The temperature was maintained at 328 K (55 °C) during the whole MD simulation process.

The Flory–Huggins parameters *χ_ij_* between the components *i* and *j* in nonpolar systems was calculated using Equation (1) if no special interactions, such as hydrogen bonding, occurred.

(1) $$\chi_{\text{ij}}=\frac{{\left(\delta_{i}-\delta_{j}\right)}^{2}V_{\mathrm{ref}}}{RT}$$

where *δ_i_* and *δ_j_* are solubility parameters of *i* and *j*, respectively, equal to the square root of cohesive energy densities; *V_ref_* is the reference volume; *R* is the gas constant; and *T* is the temperature.

The Flory–Huggins parameters *χ_ij_* of the polar components or the components with special interactions were approximately estimated using Equations (2) and (3) as follows:

(2) $$\Delta E_{\text{mix}}=\Phi_{i}{\left(\frac{E_{\mathrm{coh}}}{V}\right)}_{i}+\Phi_{j}{\left(\frac{E_{\mathrm{coh}}}{V}\right)}_{j}-{\left(\frac{E_{\mathrm{coh}}}{V}\right)}_{\mathrm{mix}}$$

(3) $$\chi_{\text{ij}}=\frac{\Delta E_{\mathrm{mix}}V_{\mathrm{ref}}}{RT}$$

where *ΔE_mix_* is the energy of mixing *i* and *j*; *Φ_i_* and *Φ_j_* are the volume fractions of *i* and *j*; ${\left(\frac{E_{\mathrm{coh}}}{V}\right)}_{i},$${\left(\frac{E_{\mathrm{coh}}}{V}\right)}_{j}$ and${\left(\frac{E_{\mathrm{coh}}}{V}\right)}_{\mathrm{mix}}$ are the cohesive energy densities of *i*, *j* and their mixtures, respectively.

DPD simulations were used to build the microstructures of ROLGs to investigate the drug and ethanol distribution in ROLGs. The coarse-grained bead volume was set at the beginning of the DPD simulation (Bowman et al., 2019; Van den Broek et al., 2018). One water molecule was considered one DPD bead. Three DPD beads and five DPD beads represented one ethanol molecule and one resveratrol molecule, respectively. A GMO molecule consisted of a total of 21 DPD beads with 5 for Fragment A and 16 for Fragment B. Interaction parameters of the different components *a_ij_* was estimated using the following Equation (4):

(4) $$a_{ij}=16.5+1.45\chi_{ij}(\rho =5,T=328\;K)$$

Where *a_ij_* is the repulsive parameter between *i* and *j* beads. Hence, Equation (4) bridges the gap between atomistic MD and DPD simulations.

For each system, the simulation box size was 20 × 20 × 20 *r*_c_ with a periodic boundary condition. The total beads were 27,302 and the spring constant C was selected as 4.0. To obtain steady results, 50,000 DPD steps were implemented with a time step of 1.0 ps.

### In vitro drug release from ROLGs

ROLGs were immersed into 200 ml of saline and then oscillated at 300 rpm at 37 °C for three parallel experiments. The administered dose was 1 g and contained 0.6 g ethanol and 4 mg resveratrol. At pre-determined time points, an aliquot (1 ml) of the mixture was withdrawn and passed through a 0.22-μm filter. Fresh saline of an equal volume was immediately used to replace the 1 ml that was withdrawn. Resveratrol in the filtrates was identified using the HPLC method (Kumar et al., 2016). The mechanism of drug release from the ROLGs was determined by inputting the release rate data into the following equations:

Zero-order model equation,

(5) $$M_{t}/M_{\infty }=kt$$

Higuchi’s square-root equation,

(6) $$M_{t}/M_{\infty }=kt^{\frac{1}{2}}$$

Ritger–Peppas’ empirical equation,

(7) $$M_{t}/M_{\infty }=kt^{n}$$

where *M_t_/M_∞_* is the fraction of drug released at time *t*; the amount of drug released at any given time (*M_t_*) was obtained from the calibration curve; the maximum amount of drug available for release (*M*_∞_) was determined by gravimetric measurements; *k* is the release rate constant of drugs from the ROLGs.

### In vitro permeation studies

Corneal tissues were harvested from euthanized male New Zealand albino rabbits (approximately 2 kg). Vertical Franz diffusion cells with an effective diffusion of 0.5 cm^2^ were used in this permeation study. The receptor compartment was filled with 15 ml of 30% ethanol/physiological saline was kept in a water bath maintained at 37 °C with constant stirring at 300 rpm. Corneal tissues were assembled between the donor and receptor cells using the corneal epithelial layer facing the donor cell. Hyaluronic acid suspensions (RHSs) or ROLGs (1 g) were placed into the donor cell, which directly was in contact with the epithelial layer of the corneal tissue. At predetermined time points (1, 3, 5, 7, 11, 19, and 24 h), 1 ml of 30% ethanol/physiological saline was withdrawn from the receptor cell and then replaced with the same volume of fresh media into the receptor cell to maintain the sink condition. The 1 ml sample was measured using the HPLC method described in the reference (Ferreira-Nunes et al., 2018; Hebert & Stöwe, 2020). Cumulative drug permeation quantities were then calculated. The slop of the linear portion of the profile represents the diffusion coefficient of the drug across the corneal tissue (*J_ss_*, μg/cm^2^/h).

### In vivo safety evaluation

Rats were randomly divided into 2 groups (6 rats/group). Animals were checked to determine if they had anterior segment diseases and/or corneal epithelial injuries before the study. Rats were treated with the ROLGs on the corneal surface once daily for 14 days were regarded as the ROLG group while those that had the resveratrol aqueous solution in 6% ethanol applied to the corneal surface on the 14th day served as the ethanol solution group. Fluorescein sodium ophthalmic test paper (Jingming New Technological Development Co., Ltd., Tianjing) was used to determine the intactness of the corneal epithelial in the two groups on the 15th day.

### Cell culture and cell viability assays

Cytotoxicity was assessed using the mitochondrial-dependent reduction of tetrazolium salt to formazan. Human corneal epithelial cell lines (HCECs) were obtained from the School of Pharmacy, Fudan University. HCECs were seeded onto 96-well culture plates (100 µl, 1 × 10^4^ cells/well) and were cultured in DMEM medium containing 10% FBS at 37 °C in a humidified incubator with 5% CO_2_ for 24 h. Afterward, media was removed from the wells, and HCECs were exposed to 100 µl of the formulation (resveratrol, blank LCG, and ROLG solution) at different concentrations (0.5, 5, 10, 20, and 50 µg/ml) for 12 h. Cells in the culture medium were used as blank control. Each group had five biological replicates. After the 12 h incubation, the media was carefully removed, and 10 µl of CCK-8 solution was added to the wells and incubated for 2 h. Cell survival rates were quantified by measuring the optical density at 450 nm using a microplate reader.

### Optical coherence tomography

Optical coherence tomography (OCT) was used to determine ROLGs retention on the surface of the rat corneas. Rats were anesthetized using an intraperitoneal injection of amobarbital (2.4 ml every rat, 100 mg/ml), and then immobilized onto the detection platform with the left eye axis upward. Anterior segment OCT images were acquired immediately and repeated every 15 min after 40 μl of eye drop formulations were dropped onto the surface of the cornea. The sweep source OCT (SS-OCT) consisted of a MEMS-based swept-source (HSL-20-100-B, central wavelength: 1310 nm; Santec Technologies, Japan), a balanced photodetector (INT-MSI-1300B; Thorlabs, Newton, NJ), a data acquisition device (ATS9350; Alarztec Technologies, Newton, NJ) and other components. The OCT provides images with approximately 12-μm axial resolution and 22-μm lateral resolution in air. The image acquisition rate was approximately 60 frames per second.

### Establishment of the alkaline injury rat cornea model

The corneas of each rat were observed under a slit lamp microscope (BOLAN, BL-66B, Shanghai, China) and those with ophthalmic diseases were excluded from the model establishment. We established the corneal alkaline burn rat model based on our previous study (Zhang et al., 2017). The ocular formulations were initially applied to rat corneas once daily until 14 days post-burn. Thirty-two burned cornea male rat models (200 ± 20 g) were equally divided into four groups. Rats that were topically administered saline onto their corneal surface were used as the control. Rats in the blank LCG group were administered 200 μl of blank LCGs, rats in the ROLG group were administered 200 μl of ROLGs, and rats in the RHS group were administered 200 μl of RHSs.

### Corneal vessel visualization assays

CNV growth in each group was observed using a slit-lamp microscope imaging system on days 7, 10, and 14. Neovascularization was quantified by measuring the length of the vessels (from the limbus to the tip of the vessels) using the measuring tool in Image J (NIH, US) before determining the CNV area.

### CNV histology

Rats were sacrificed on day 14, and the corneal tissues were harvested and fixed in 4% paraformaldehyde after the conjunctiva and sclera were removed. Paraffin-embedded corneal tissues were sectioned and stained with hematoxylin and eosin. Histological sections of the corneal tissues were examined under a Nikon Eclipse microscope (Tokyo, Japan) to observe changes in the tissue structure.

### Measurement of VEGF expression levels

Corneal tissues embedded in paraffin were deparaffined in xylene and rehydrated with ethanol. The tissues were then immersed in ethylene diamine tetraacetic acid antigen retrieval solutions (pH 8.0) and antigens were removed after microwave heating for 15 min. Tissues were washed with water for 5 min and then processed with hydrogen peroxide solution (3%, 30 μl) to remove endogenous peroxidases. Tissues were then incubated with anti-VEGF primary antibody diluted in PBS (pH = 7.4) for 30 min at room temperature, and then washed and incubated with secondary antibody for 30 min at room temperature. Immunohistochemical staining was performed based on the manufacturer’s instruction. Tissues were then observed for positive VEGF staining using a microscope (Nikon, E100, Tokyo, Japan).

### Statistical analysis

Student’s *t*-test and one-way ANOVA were performed using the SPSS 21.0 software (SPSS Inc.). Data were expressed as mean ± standard deviation, *p* < .05 was considered statistically significant.

## Results and discussion

### Formulation and characteristics of ROLGs

Various ROLG component ratios were investigated. The fraction of purified water was constant and the ratio of GMO/ethanol was adjusted. LCGs were obtained by adding ethanol organic solvents (propylene glycol, ethanol, polyethylene glycol) (Sagitani & Komoriya, 2015; Abbas et al., 2018). However, ethanol at the interface of GMO/water reduced the surface tension of lipid crystals, hence, were vulnerable to phase transition with the change in ethanol. When the ethanol ratio was reduced, the product was a homogeneous stiff transparent gel but was a slightly opaque solution with low viscosity when the ethanol ratio was increased. A homogeneous lamellar crystal gel (LCG) with appropriate viscosity was obtained when the ratio of GMO/ethanol/purified water was 6:0.6:3.

We observed high resveratrol solubility in the ROLGs (4.4 mg/g). No precipitates were observed by the naked eye and no solids were observed under a microscope. The high solubility was very much related to GMO inhibiting crystallization. Furthermore, high drug concentrations in the formulations were likely to facilitate drug penetration through the corneal barrier (Li et al., 2018).

**Figure 2.**
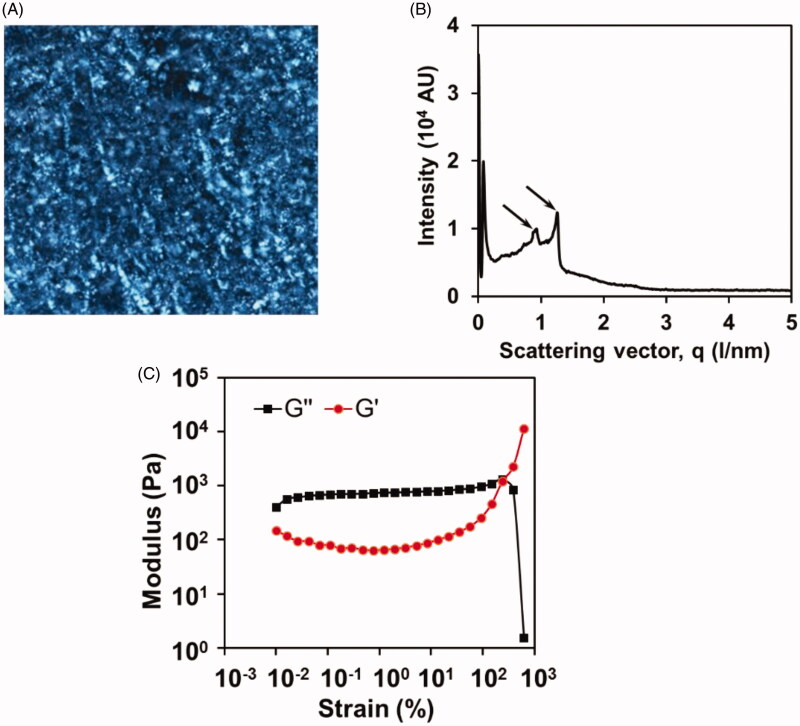
(A) PLM images of ROLGs at room temperature (400×). (B) SAXS diffraction pattern of ROLGs. (C) Storage modulus (G′) and Loss modulus (G′′) as a function of angular frequency for ROLGs.

The ROLGs were optically anisotropic with a streak texture under PLM (Figure 2(A)), suggesting the typical characteristics of lamellar crystalline gels. Moreover, SAXS scatterings (Figure 2(B)) showed two typical lamellar peaks with a defined 3:4 spacing ratio. This further illustrated the lamellar crystal structure of the ROLGs.

Oscillatory strain sweep reflects the linear viscoelastic domain of the gels (Carvalho et al., 2016). The G′′ and G′ of the ROLGs remained constant in the strain range of less than 100%, indicating the complete structure of the hydrogels even though a large deformation was applied (Figure 2(C)). After the strain exceeded 100%, G′′ decreased dramatically, suggesting the collapse of the ROLG structure (Marlow et al., 2018; Chen et al., 2019).

### Inhibition of resveratrol crystallization in the ROLGs

**Figure 3.**
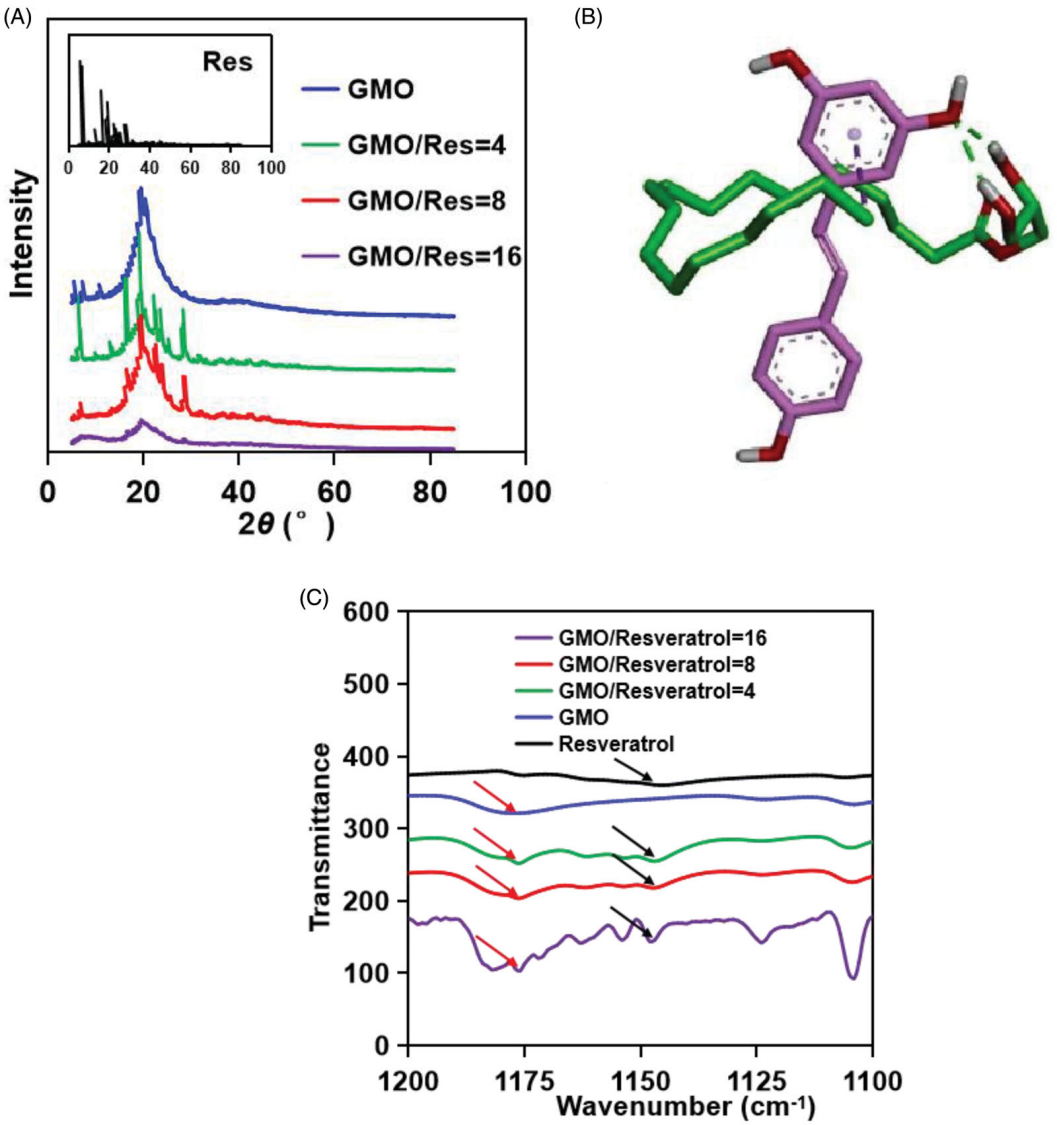
(A) PXRD patterns of pure resveratrol (black), pure GMO (blue), and GMO/resveratrol at weight ratios of 4 (green), 8 (red), and 16 (purple). (B) Chemical structures with proposed hydrogen-bonding between GMO and resveratrol. (C) FTIR absorption spectra of pure resveratrol (black), pure GMO (blue), and GMO/resveratrol at weight ratios of 4 (green), 8 (red), and 16 (purple). The absorption peaks of the hydroxyl group of resveratrol (black arrow) and GMO (red arrow) were shifted at different ratios.

The PXRD pattern of a crystalline material typically exhibits sharp crystalline peaks (Ikuta et al., 2015), while an amorphous material shows a broad background described as a halo pattern (Abbas et al., 2018). Some strong diffraction peaks were observed in the PXRD pattern for resveratrol at approximately 8°–27°, demonstrating that resveratrol was a crystalline drug (Figure 3(A)). Similarly, GMO had a sharp peak at 22.4°, which suggested GMO was a crystalline material. The PXRD patterns of the complexes at different ratios revealed a tendency for the characteristic diffraction peak intensity of the drug to become weak as the ratio of GMO/resveratrol increased. When the weight ratio of GMO/resveratrol was equal to 16, the crystal diffraction peaks of resveratrol disappeared. This indicated that resveratrol in an amorphous state was dispersed in the GMO/complexes.

### Intermolecular hydrogen bonds between GMO and resveratrol

Molecular docking was used to observe intermolecular interactions (Prior & Oganesyan, 2017). The hydroxyl group of GMO could form hydrogen bonds with the hydroxyl group of resveratrol, the binding energies of which were −1.2 kcal/mol in total (Figure 3(B)). Our results indicated that GMO may inhibit resveratrol crystallization via hydrogen bonding between GMO and resveratrol.

The absorption peak at 1145 cm^−1^ in the resveratrol spectrum was attributed to the hydroxyl group (Table 1). However, the peak showed a blue-shift to 1147 and 1148 cm^−1^ in the GMO/resveratrol complex at various ratios of 4–16 (Figure 3(C)). Additionally, the GMO peak showed a red-shift from 1177 to 1176 cm^−1^ in the GMO/resveratrol complex. This signified considerable hydrogen-bonding between the hydroxyl groups of GMO and resveratrol.

**Table 1.** Peaks in FT-IR spectra for the hydroxyl group of GMO and resveratrol at different ratios.

	Hydroxyl group of GMO (cm^−1^)	Hydroxyl group of resveratrol (cm^−1^)
GMO	1177	
Resveratrol		1145
GMO/resveratrol = 4	1176	1147
GMO/resveratrol = 8	1176	1147
GMO/resveratrol = 16	1176	1148

In summary, PXRD, molecular docking, and FT-IR spectra showed remarkably hydrogen bonding between GMO and resveratrol, leading to the inhibition of resveratrol crystallization in ROLGs and dramatically increasing the solubility of resveratrol.

### Flory–Huggins parameters

MD simulations are usually performed to estimate Flory–Huggins parameters (Mao et al., 2019). Herein, Equation (1) was only used to calculate the Flory–Huggins parameters between the components without hydrogen bonding, such as between water and Fragment B (Luo & Jiang, 2012). Equations (2) and (3) were applied to components with hydrogen bonding such as between water and Fragment A.

Absolutes of the Flory–Huggins parameters (*χ*) less than 0.5 suggests that the binary components are miscible, while negative denotes attraction of binary components, which were observed for all Flory–Huggins parameters of the components in ROLGs. The *χ*_H2O/A_ was predicted to be −3.04 (Table 2), suggesting that Fragment A was insoluble in H_2_O and that there was a certain amount of attraction between them. The estimated *χ*_H2O/B_ equal to 5.1 indicated that Fragment B was hydrophobic, which made up the lipid lyophobic region of the lipid crystal. Resveratrol/Fragment A was immiscible, however, a mutual attraction was present, while Resveratrol/Fragment B was miscible based on the Flory–Huggins parameters.

**Table 2.** Molecular parameters in MD simulations at 328 K.

Components	Number of molecules	*δ* (J/cm^3^)^0.5^	*χ*	*a*
H_2_O	700	43.5		
Ethanol	200	24.6		
Resveratrol	80	26.4		
Fragment A^a^	80	28.7		
Fragment B^a^	80	16.2		
H_2_O/resveratrol	880/4		−0.75	15.4
H_2_O/fragment A	17/356		−3.04	12.1
H_2_O/fragment B^b^	17/356		5.10	23.9
H_2_O/ethanol	166/13		−0.07	16.4
Resveratrol/fragment A	2/171		−1.22	14.9
Resveratrol/fragment B	2/171		0.07	16.6
Resveratrol/ethanol	2/130		0.34	17.0
Fragment A/fragment B	12/12		1.03	18.0
Fragment A/ethanol	17/13		−0.40	16.1
Fragment B/ethanol	17/13		0.48	17.2

^a^ Refer to Figure 1 for the meanings of Fragments A and B of GMO. ^b^The Flory–Huggins parameters of H_2_O/Fragment B are presented as *χ_B/H2O_*. Other *χs* are present as the similar mode.

### DPD modeling of ROLGs

**Figure 4.**
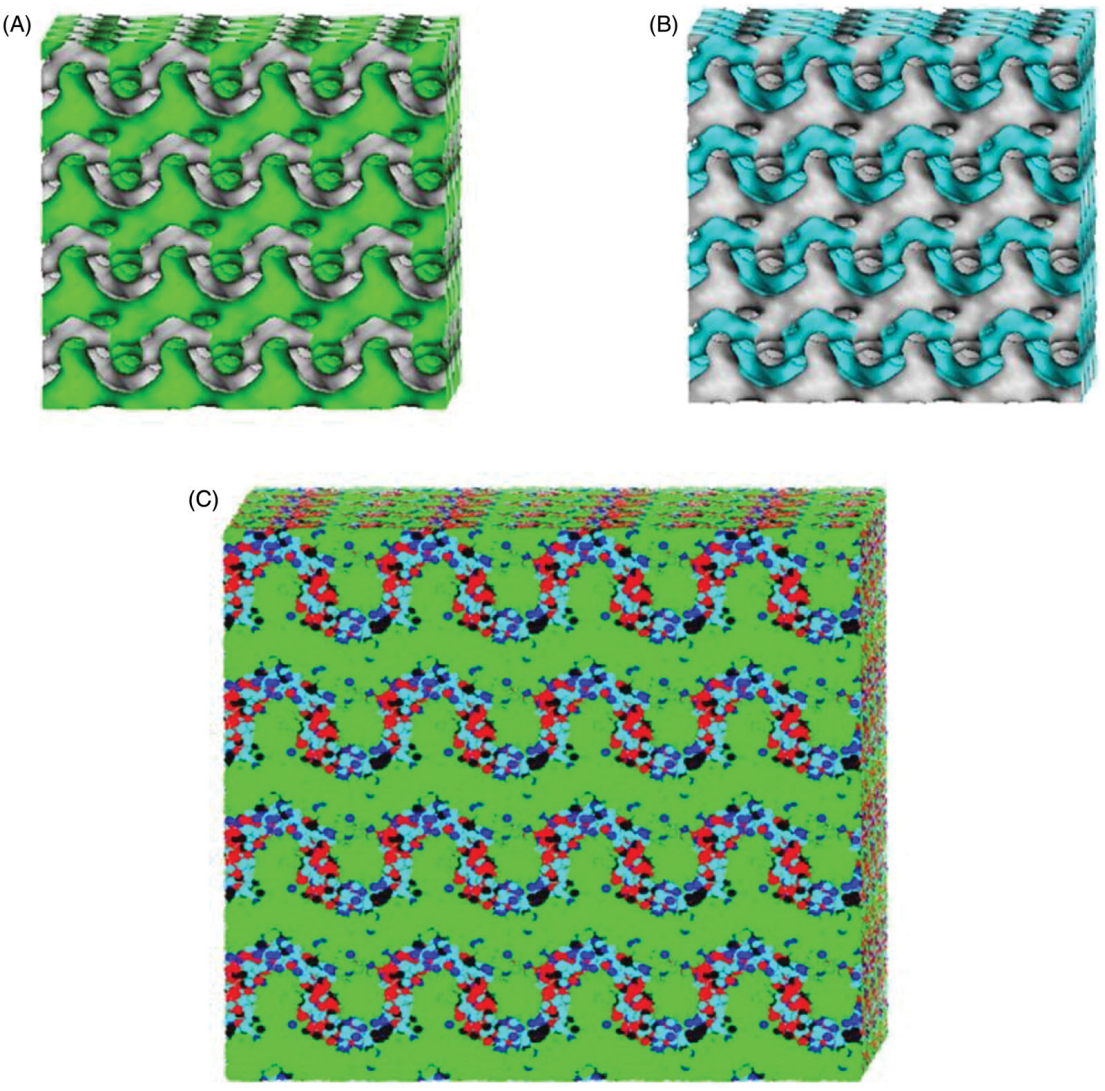
DPD simulation characteristics of ROLGs. Fragment B (A) and water (B) iso-density surfaces of ROLGs. ‘Dot-and-line’ model (Fragment A, Fragment B, resveratrol, ethanol, and water particles are depicted in red, green, black, blue, and light blue, respectively) (C).

Iso-density surfaces reflect the internal structure of the ROLGs. The inner structure of the ROLGs contained bi-continuous parallel channels of lipid and water (Figure 4(A,B)), and was typical of lamellar crystallines (Bartolini et al., 2019).

The ROLGs exhibited complex spatial organization, where the hydrophilic groups of GMO (Fragment A) were inward, while the hydrophobic group (Fragment B) was outward as shown in the ‘dot-and-line’ model in Figure 4(C). Ethanol has been widely used in ocular surface surgeries and for the treatment of corneal diseases. However, brief exposure of the corneal surface to ethanol has long-term effects by disrupting the integrity of the corneal epithelium and inducing inflammation. Ethanol distribution in the ROLGs was mainly localized at the interface between the two channels as stated above. This observation was consistent with the results of a previous publication (Efrat et al., 2008). Ethanol in the ROLGs may only indirectly get in contact with the corneal surface due to the typical structure of lamellar crystals. This reduces the harmful effects of ethanol on corneal tissues. Similarly, resveratrol was also distributed around the interface between the two channels due to the hydrogen bonding between GMO-resveratrol and drug hydrophobicity. These findings indicate that the release of resveratrol may depend on ROLG degradation.

### Sustained-release of resveratrol from ROLGs

**Figure 5.**
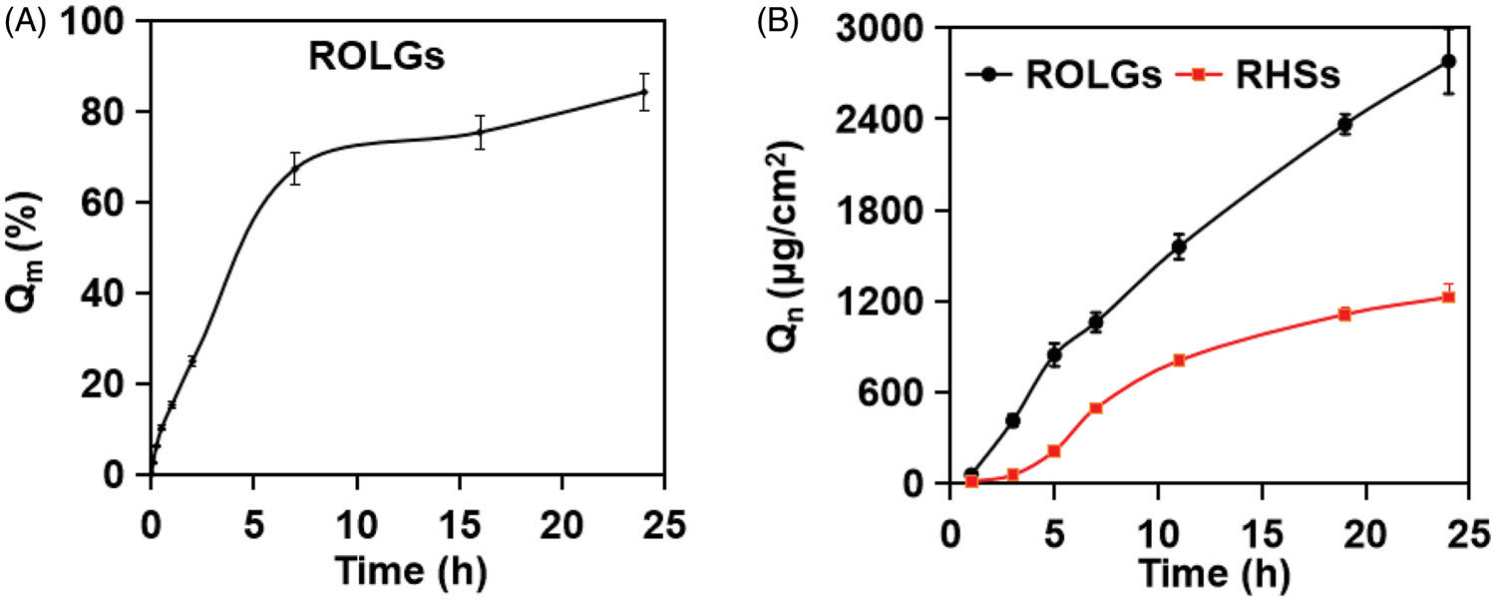
Resveratrol release and permeation from ROLGs. (A) *In vitro* resveratrol release profile of ROLGs. (B) *In vitro* resveratrol cornea permeation profiles of ROLGs and RHSs.

ROLGs could provide a mechanism for the sustained release of resveratrol (Figure 5(A)). At 30 min post-dissolution, 10% cumulative drug release was achieved. Additionally, at 7 h post-dissolution, 67% cumulative drug release was achieved. Stable drug release from ROLGs could facilitate drug permeation across the corneal (Fonseca-Santos et al., 2017). A linear relationship was found between drug release and the third power of time. This indicated that release kinetics could be explained by the Ritger–Peppas’ equation (Table 3). Resveratrol release was mainly through erosion control, which was supported by the above DPD simulation.

**Table 3.** Simulations of drug release from ROLGs.

Simulation model	Parameter	
	Release rate constant (*k*)	Correlation coefficient (*r*)
Zero-order equation	0.036	.840
Higuchi’s equation	0.192	.955
Ritger–Peppas’ equation	0.630	.980

### Improved resveratrol permeation across the cornea by ROLGs

A Franz diffusion cell system was used to investigate corneal permeability of resveratrol in different formulations. We observed that ROLGs permeated far better compared to RHSs (Figure 5(B)). We found that 412 μg of resveratrol permeated from ROLGs at 3 h compared to only 59 μg from RHSs. The permeated amounts were 845 and 213 μg at 5 h, and 1557 and 807 μg at 11 h, respectively. The diffusion coefficient of ROLGs (*J_ss_* = 115 ± 48 μg/cm^2^/h) was demonstrably higher compared to RHSs (*J_ss_* = 56 ± 11 μg/cm^2^/h). These results indicated that ROLGs significantly enhanced corneal penetration of resveratrol compared to the control dosage form. GMO may enhance drug permeation through the cornea by increasing the fluidity of corneal lipids (Efrat et al., 2008). Highly efficient resveratrol permeation from ROLGs is likely to inhibit CNV.

### In vivo safety of ROLGs

**Figure 6.**
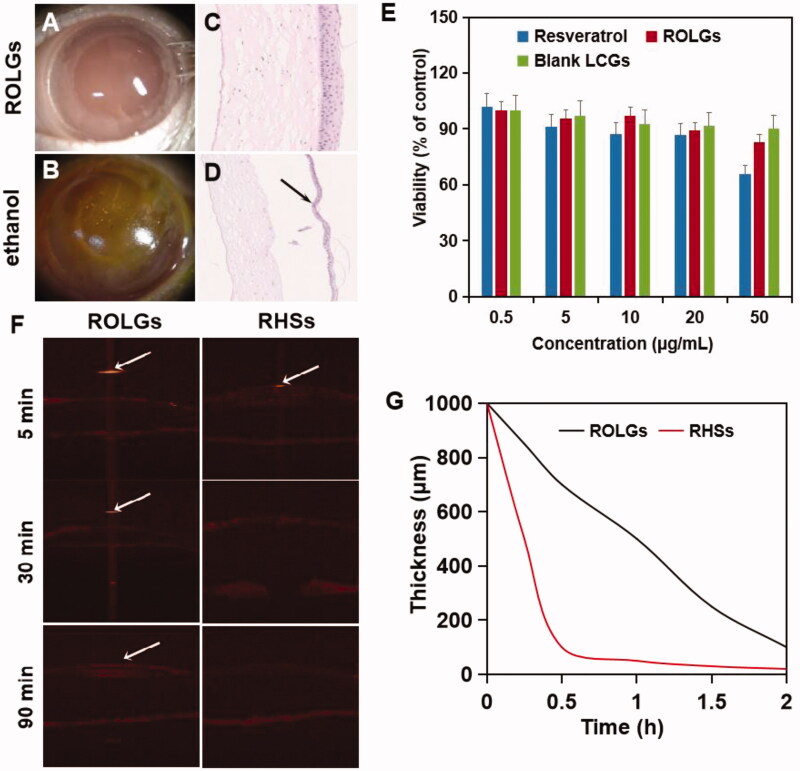
Images of stained corneal epithelial after ROLG (A) and ethanol solution (B) exposure. Corresponding pathological sections after ROLG (C) and ethanol solution (D) exposure. Black arrows indicate severe corneal epithelial damage. (E) Results of CCK-8 evaluation (*n* = 5). Cell viabilities of human corneal epithelial cells after exposure to resveratrol solution, blank LCG solution, and ROLG solution. Blank control was considered 100%. (F) Typical representative OCT images used to quantify eye gel layer thickness. (G) Preparation thickness in the center of the cornea at different time points.

The integrity of the corneal epithelium was measured using the sodium fluorescein test (Bandamwar et al., 2014). Corneas in the ROLG group were glossy with no staining observed (Figure 6(A)), indicating no damage to the corneal epithelium. However, the integrity of the corneal epithelium in the resveratrol solution group was damaged due to ethanol exposure (Figure 6(B)). Corneal tissues in the ROLG group were intact, and the cells on each layer were arranged neatly (Figure 6(C)). However, corneal epithelial exposed to resveratrol solution was damaged and was characterized by the disorder to the stromal layer (Figure 6(D)). These results demonstrated that ROLGs was a safe ocular delivery system.

The amount of ethanol in ophthalmic preparations are regulated due to its adverse effects on corneal tissues. This significantly limits drug loads. The ethanol in ROLGs does not directly come in contact with the corneal tissues due to the unique lamellar structure of LCGs. Hence, ROLGs not only can achieve high drug loads but ensures that the adverse effects of ethanol are limited.

### Cell viability

HCEC cell viability after exposure to resveratrol solution, blank LCG solution, or ROLG solution is shown in Figure 6(E). The viability of HCECs reduced with increasing sample concentration. Resveratrol solution and ROLGs did not significantly affect cell viability, except in high concentration (50 μg/ml). We also did not detect cytotoxic effect at the other two solutions, indicating that resveratrol, blank LCG, ROLG were safe for drug delivery.

### Long-term retention of ROLGs on the ocular surface

ROLG retention on the ocular surface was evaluated by thickness comparison using real-time OCT (Aumann et al., 2019). An aliquot (50 μl) of the two types of eye gel was pipetted onto the surface of anesthetized rat eyes, and then clearance time was measured. The mean thickness of the ROLG was 1000 μm at 0 min and reduced to 50 μm at 90 min post-application (Figure 6(G)). A thin layer of ROLG remained on the surface of the rat cornea after 90 min (Figure 6(F)). However, RHS was cleared within the first 30 min and was no longer observable at 90 min post-application (Figure 6(F,G)). These results demonstrated that LCGs had long drug retention on the corneal surface.

### Efficacy of ROLGs on CNV

**Figure 7.**
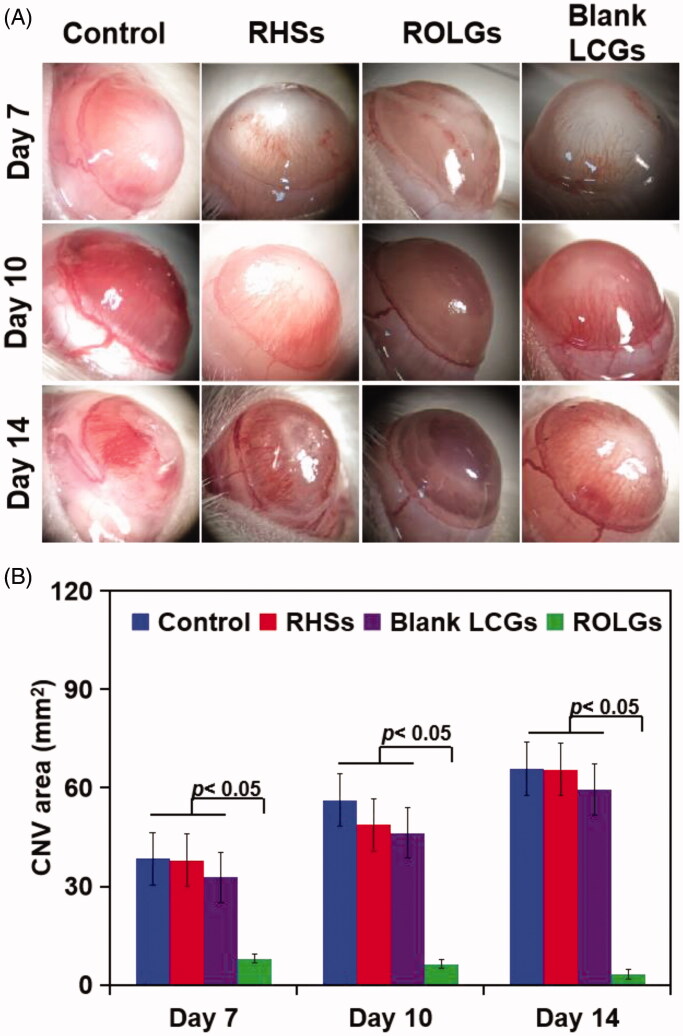
(A) Development of corneal neovascularization. Representative photographs of the four treatment groups: Control, RHSs, ROLGs, and Blank LCGs. (B) Graphs denoting the mean corneal neovascularization area in the treatment groups at14 days post-injury, *p < .05 vs Control, *p < .05 vs RHSs, *p < .05 vs Blank LCGs.

CNV is a major cause of blindness during corneal wound healing (Yureeda et al., 2010; Zhang et al., 2018). Significant neovascularization was observed on the corneas of each group in the first few days (Figure 7(A)). Rats in the ROLG group had better therapeutic efficacy compared to the other treatment groups at the same time points measured (Figure 7(A)). The areas of new vessels for each group increased at day 14 post-injury but to different extents for the different groups (Figure 7(B)). The difference was statistically significant between the ROLG group and the control (*p* < .05) when measured at the same time points. This strongly suggested that ROLG inhibited CNV during the process of corneal wound healing.

### ROLGs promote corneal epidermal recovery

**Figure 8.**
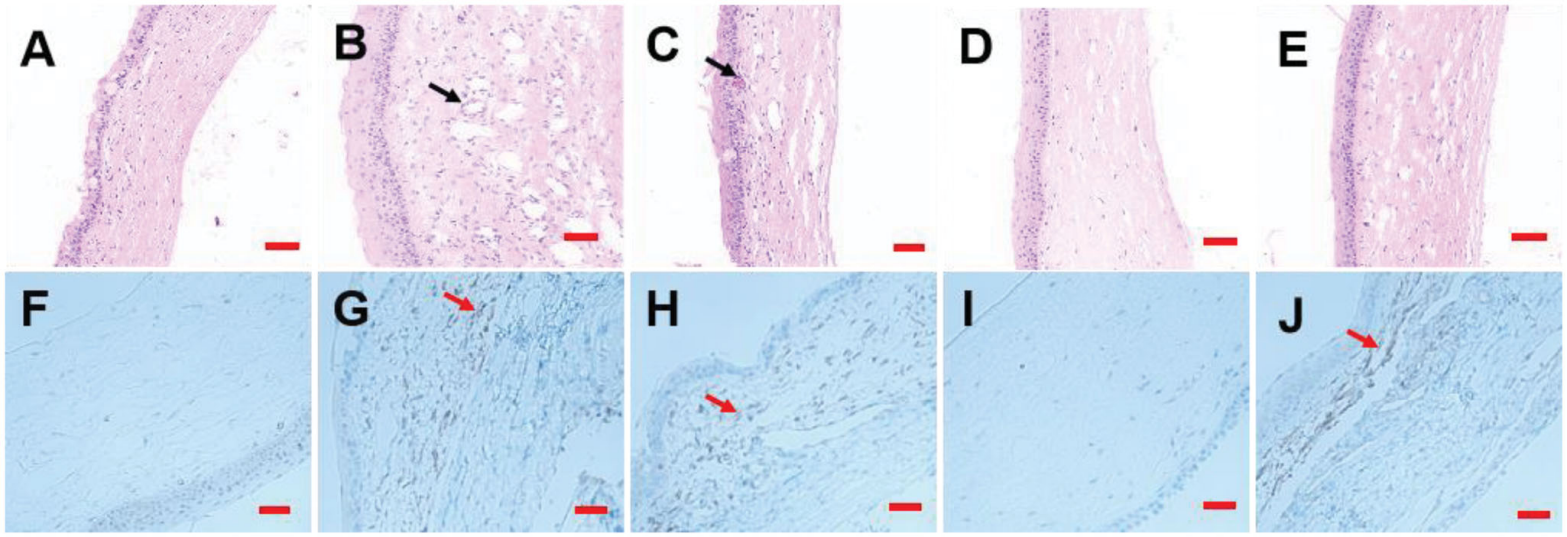
Effects of resveratrol on corneal epidermal recovery. H&E images of corneal tissues in the healthy group (A), the control group (B), the RHS group (C), the ROLG group (D), and the blank LCG group (E). Black arrows indicate neovascularization. Immunohistochemistry analysis of VEGF expression in the healthy group (F), the control group (G), the RHS group (H), the ROLG group (I), and the blank LCG group (J). Red arrows represent VEGF expression in the corneal tissues. Scale bars indicate 100 μm.

Corneal epidermal recovery is a critical step in corneal wound healing, and complete corneal epidermal recovery contributes to vision recovery (Huang et al., 2015). Compared to healthy corneal tissues (Figure 8(A)), HE images of corneal tissues in the control, RHS, and blank LCG group had disorganized matrix fibers, edema, hyperemia, numerous inflammatory cells, and several neovascularizations in the cornea that formed vessel nets (Figure 8(B,C,E)). In comparison, corneal tissues in the ROLG group showed complete corneal epidermal recovery characterized by organized matrix fibers with only a few inflammatory cells (Figure 8(D)). These results demonstrated that ROLGs would significantly enhance corneal epidermal recovery.

### ROLGs reduces VEGF expression levels in the cornea

The imbalance between angiogenic and antiangiogenic factors is important for CNV pathogenesis (Zhong et al., 2018). VEGF is the most essential angiogenic factor responsible for the growth of new vessels. It is secreted by corneal epithelium cells and then sequestrated by the basement membrane in physiological conditions (Cakmak et al., 2018; Fu & Xin, 2019). The expression levels of VEGF in the control group were higher compared to the healthy group (Figure 8(F,G)), indicating that corneal alkali burns increased VEGF expression, which thereby induced CNV. Resveratrol is a highly effective anti-VEGF agent against CNV (Hu et al., 2019). Ocular drug delivery has been a challenge due to physiological eye obstacles such as blinking and the production of tears. This makes it difficult to attain an effective drug concentration within the target eye tissues (Huang et al., 2018). Rats in the RHS and blank LCG groups had similar VEGF expression levels compared to the control group as shown by immunohistochemistry (Figure 8(G,H,J)). This demonstrated that the permeability of RHS was inefficient due to the barrier properties of corneal epithelium. VEGF expression levels in the ROLG group were significantly lower compared to the control group (Figure 8(I)). These results demonstrated that ROLGs improved corneal epidermal recovery by inhibiting VEGF expression due to its high permeability.

## Conclusion

Resveratrol has been demonstrated to be a highly effective anti-VEGF agent. VEGF is a critical pathogenic factor for CNV. High drug hydrophobicity and physiological eye obstacles make it difficult to penetrate across the corneal epidermis and inhibit CNV. In this study, we demonstrated that a lamellar crystalline gel formulation of resveratrol, i.e., ROLGs, had robust retention on the ocular surface with the high drug loading ability. Furthermore, ROLGs enhanced drug permeability across corneal tissues. More importantly, ROLGs are relatively easily administered and safe for continuous delivery of resveratrol, resulting in high anti-VEGF to suppress CNV. Therefore, LCG is an ideal ocular delivery system for resveratrol and a potential treatment strategy for CNV.
